# HLA-B*15:21 and carbamazepine-induced Stevens-Johnson syndrome: pooled-data and *in silico* analysis

**DOI:** 10.1038/srep45553

**Published:** 2017-03-30

**Authors:** Kanoot Jaruthamsophon, Varomyalin Tipmanee, Antida Sangiemchoey, Chonlaphat Sukasem, Pornprot Limprasert

**Affiliations:** 1 Department of Pathology, Faculty of Medicine, Prince of Songkla University, Songkhla 90110, Thailand; 2 Department of Biomedical Sciences, Faculty of Medicine, Prince of Songkla University, Songkhla 90110, Thailand; 3 Division of Pharmacy, Songklanagarind Hospital, Faculty of Medicine, Prince of Songkla University, Songkhla 90110, Thailand; 4 Division of Pharmacogenomics and Personalized Medicine, Department of Pathology, Faculty of Medicine Ramathibodi Hospital, Mahidol University, Bangkok 10400, Thailand; 5 Laboratory for Pharmacogenomics, Somdech Phra Debaratana Medical Center (SDMC), Faculty of Medicine Ramathibodi Hospital, Mahidol University, Bangkok 10400, Thailand

## Abstract

HLA-B*15:02 screening before carbamazepine (CBZ) prescription in Asian populations is the recommended practice to prevent CBZ-induced Stevens-Johnson syndrome (CBZ-SJS). However, a number of patients have developed CBZ-SJS even having no HLA-B*15:02. Herein, we present the case of a Thai patient who had a negative HLA-B*15:02 screening result but later developed CBZ-SJS. Further HLA typing revealed HLA-B*15:21/B*13:01. HLA-B*15:21 is a member of the HLA-B75 serotype and is commonly found in Southeast Asian populations. Based on this case, we hypothesised that if all HLA-B*15:02 carriers were prevented from CBZ prescription, another common HLA-B75 serotype marker would show its association with CBZ-SJS. To test this hypothesis, we pooled data from previous association studies in Asian populations, excluded all cases with HLA-B*15:02, and analysed the association significance of HLA-B75 serotype markers. A significant association was found between CBZ-SJS and HLA-B*15:21 and HLA-B*15:11. We also applied an *in silico* analysis and found that all HLA-B75 serotype molecules shared similar capability in binding the CBZ molecule. In summary, this report provides the first evidence of a positive association between HLA-B*15:21 and CBZ-SJS and the first *in silico* analysis of CBZ binding sites and details of the molecular behaviour of HLA-B75 molecule to explain its molecular action.

## Introduction

*Human Leukocyte Antigen* (HLA) -B*15:02 is a genetic marker strongly associated with carbamazepine (CBZ)-induced Stevens-Johnson syndrome and toxic epidermal necrolysis (CBZ-SJS) in Asian populations^1^. Screening for HLA-B*15:02 before CBZ prescription has proven to prevent CBZ-SJS^2^. However, a number of cases of patients who developed CBZ-SJS while having no HLA-B*15:02 have been reported^3^,^4^,^5^,^6^. Herein, we report the case of a Thai patient who developed CBZ-SJS while screening negative for HLA-B*15:02 and was later found to have HLA-B*15:21, an HLA-B75 serotype marker similar to HLA-B*15:02, B*15:11, and B*15:08.

We hypothesised that if all HLA-B*15:02 carriers were prevented from CBZ prescription, another common HLA-B75 serotype marker – especially HLA-B*15:21 – would reveal its nature in causing CBZ-SJS. To test this hypothesis, we reviewed published association studies in Asian populations (excluding Japanese and Korean studies, as these populations have been found to have no association with HLA-B*15:02 7,8), pooled genotype data, excluded all cases and controls with HLA-B*15:02, and then analysed the association between HLA-B75 serotype markers and CBZ-SJS. Since the serotype manifests in a certain protein form, a tertiary structure of the protein member is necessary for detailed investigation. In our study, we did not only construct a tertiary structure but also performed an *in silico* analysis to evaluate all HLA-B75 structures and the molecular binding between the CBZ molecule and all HLA-B75 serotype molecules compared with the HLA-B*15:01 molecule.

## Results

### Case report

A 14-year-old male patient presented with muscle spasms of his left arm and leg for 6 months. His symptoms were aggravated when he moved quickly. He was diagnosed with paroxysmal kinesigenic dyskinesia and was screened for HLA-B*15:02 by a validated in-house method^9^. HLA-B*15:02 screening was negative and the patient was prescribed CBZ 200 mg per day. This patient had neither previous history of food nor drug allergy and had never been prescribed CBZ.

After taking the CBZ for 14 days, the patient developed a maculopapular rash at both arms and trunk, and also had injected eyes and oral ulcers. He was diagnosed with CBZ-SJS, at which time the CBZ was discontinued and he was admitted into the hospital. His symptoms resolved 14 days after the CBZ was discontinued, and he was prescribed topiramate 50 mg per day. A DNA sample was re-examined for HLA typing by the PCR-SSOP method (Luminex^®^IS 100, USA), which revealed HLA-A*02:03/A*24:02 and HLA-B*13:01/B*15:21.

### Pooled-data analysis

A search of the HLA and Adverse Drug Reaction Database (accessed on 3 June 2016) found 33 studies evaluating the association between *HLA-B* alleles and antiepileptic drugs^10^. Among these studies, 4 studies did not involve an association between *HLA-B* markers and CBZ-SJS, 9 studies involved European, Japanese, Korean, and Canadian populations, 15 studies had insufficient data for analysis, and the full text of 1 study was not accessible. Finally, only 4 case-control studies were eligible for the pooled-data analysis.

The pooled data of the selected studies are shown in Table 1. Among the total of 108 CBZ-SJS cases, 13 cases had no HLA-B*15:02. Only 4 cases were found with the HLA-B75 serotype. There were 225 tolerant controls who had no HLA-B*15:02. The only reported tolerant control with the HLA-B75 serotype was found to have HLA-B*15:21 in the Hong Kong Han Chinese study^4^.

The pooled-data analysis revealed a significant association between CBZ-SJS and both HLA-B*15:21 (Odds ratio = 40.73, 95% CI = 3.43–484.22, *P* = 0.003) and HLA-B*15:11 (Odds ratio = 54.12, 95% CI = 2.10–1397.09, *P* = 0.016) (Table 2). However, we were unable to evaluate any possible associations between CBZ-SJS and HLA-B*15:08 and other common *HLA-B* alleles, as negative association controls, because of insufficient genotype data in the tolerant control group.

### *In silico* analysis

**Figure 1:**
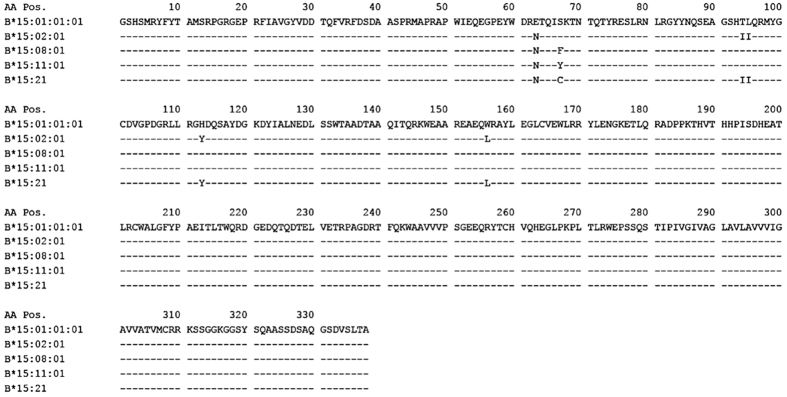
Alignment of the amino acid sequences of HLA-B*15:01, B*15:02, B*15:08, B*15:11, and B*15:21. The data were obtained from the Sequence Alignment Tool found in the IMGT/HLA database version 3.25.0 (accessed on 8 August 2016).

To understand how base changes affect a protein structure and CBZ reacts to the protein structure, a sequence alignment was performed among all HLA-B75 serotype members of interest to evaluate the possibility of modelling the tertiary structures and investigating the molecular information. The sequence alignment, Fig. 1, shows a high similarity among the proteins of interest with respect to HLA-B*15:01, in which the tertiary structure is available in the experimental RCSB PDB database (pdb code 1XR9)^11^. At least 95% of the amino acids were conserved, clearly seen from Fig. 1.

**Figure 2:**
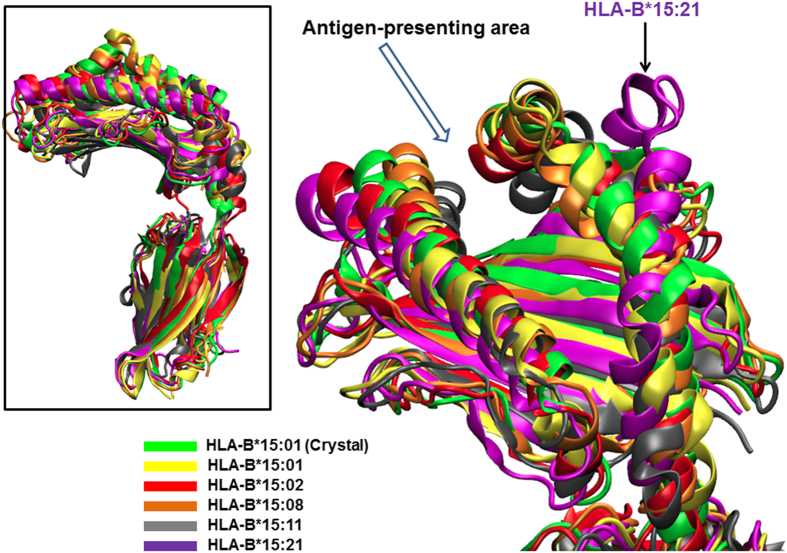
Conformation comparison of HLA-B*15:01 and HLA-B75 protein species. All simulated structures were aligned with respect to the HLA-B*15:01 crystal structure to visualise the effects due to amino acid changes on protein conformation. The protein conformations at the antigen-presenting area are similar to the HLA-B*15:01 crystal structure (green ribbons), except a larger gap was found in HLA-B*15:21 (purple ribbons with arrow indicated).

According to the sequence alignment, the other HLA-B75 protein structures can be successfully modelled and simulated using molecular dynamics (MD) simulation to assess the protein conformational details. From our results (Fig. 2), MD sampling showed that a protein conformation of the antigen-presenting area in the HLA-B75 protein remains similar among species except for HLA-B*15:21. In Fig. 2, the domain of HLA-B*15:21 can be seen to be larger, compared to other HLA-B75 proteins and HLA-B*15:01 crystal structure.

**Figure 3:**
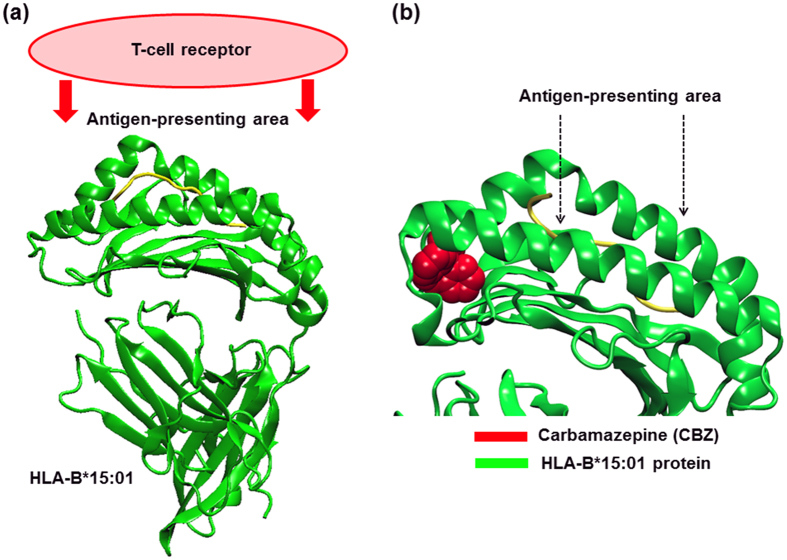
Possible binding sites of CBZ in HLA-B*15:01. (**a**) HLA-B*15:01 functions via binding the T-cell receptor protein using the antigen-presenting area. The green ribbons represent the HLA-B*15:01 protein structure. (**b**) The two arrows point at the binding groove of the HLA-B*15:01 molecule, also defined as an antigen-presenting area. A possible binding site was determined by a docked CBZ conformation (red) with the lowest binding energy. In HLA-B*15:01, CBZ possibly binds the protein, however, the binding interactions are caused from the amino acid excluding from the antigen-presenting area. In other words, CBZ could bind HLA-B*15:01 but it could not incorporate into the antigen-presenting area.

**Figure 4:**
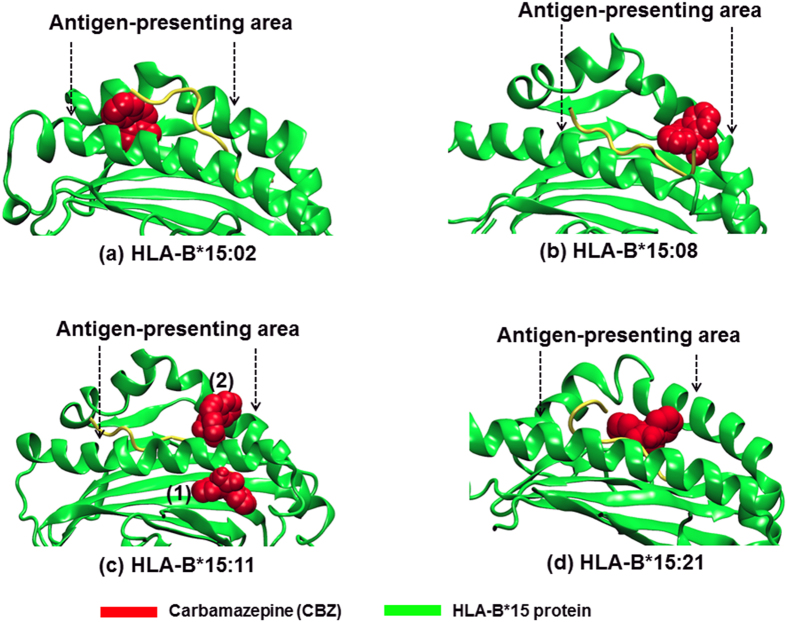
Possible binding site of CBZ in HLA-B75 protein structure. The green ribbons represent the HLA-B75 protein structure, with the two arrows pointing to the binding groove of CBZ (red), also defined as an antigen-presenting area. In (**a**) HLA-B*15:02, (**b**) B*15:08, and (**d**) B*15:21, CBZ possibly binds the protein using interactions from the antigen-presenting area. In (**c**) HLA-B*15:11, the best conformation (1) is located outside the antigen-presenting area, but the second best conformation (2) is located at the antigen-presenting area.

Apart from the protein conformation, the selectivity of CBZ binding to the HLA-B75 family was evaluated using the molecular docking approach. In this study, we illustrate the possible CBZ binding sites on HLA-B75 protein on the basis of binding energy. The sites with a lower binding energy (more negative number) indicate a higher possibility and more favourable binding preference. Table 3 shows the binding energy results from a docking study between CBZ and HLA-B*15:01 and the various HLA-B75 members. In addition, the structure of the CBZ-HLA-B molecule complex from the molecular docking approach is also illustrated to identify the possible CBZ binding sites in Figs 3 and 4.

## Discussion

Even though HLA-B*15:02 screening is effective in preventing CBZ-SJS, there have been a number of reported cases of people who developed CBZ-SJS even having neither HLA-B*15:02 nor the other known associated markers, HLA-A*31:01 and B*15:11^3^,^4^,^5^,^6^. Our patient had screened negative for HLA-B*15:02, but we later found HLA-B*15:21, another HLA-B75 serotype marker that could be a possible cause of CBZ-SJS. Similarly, the pooled data also included 4 CBZ-SJS cases who had no HLA-B*15:02 but other HLA-B75 serotype makers: 2 cases with HLA-B*15:21 and 1 each of HLA-B*15:08 and B*15:11 (Table 1).

As HLA frequencies vary greatly among populations, the statistical significance of the association studies has also varied regarding the *HLA-B* allele frequencies. For example, HLA-B*15:21 is common in Southeast Asian and neighbouring populations whose HLA-B*15:02 frequency is also high^10^,^12^. It is not surprising, then, that to date, association studies have found no connection between HLA-B*15:21 and CBZ-SJS, as there are only a small number of cases with HLA-B*15:21 as the large majority of cases have HLA-B*15:02. Only after the HLA-B*15:02 cases were excluded and cases from a number of studies were pooled did we find statistical significance for HLA-B*15:21. Since this pooled data included only a limited number of CBZ-SJS cases without the HLA-B*15:02 allele, further case-control studies from real-world populations are needed to confirm the associations between CBZ-SJS and the HLA-B*15:21, as well as other HLA-B75 serotype markers.

Although it is a significant new finding, the positive association between CBZ and HLA-B*15:21 is not an unexpected finding, since the HLA-B*15:21 marker is a member of the HLA-B75 serotype group, similar to HLA-B*15:02 and B*15:11. These HLA-B75 serotype members, including HLA-B*15:08, share the 63^rd^ arginine residue (Fig. 1) which is critical for the formation of the drug-HLA molecule complex by a direct pharmacological interaction and is able to induce an *in vitro* lymphocyte cytotoxic response to CBZ^13^.

However, only HLA-B*15:02 and B*15:11 have been proven to be associated with CBZ-SJS in real-world association studies^3^,^4^,^5^,^6^,^7^,^8^. In this study, we provide the first positive association between CBZ-SJS and HLA-B*15:21 but not HLA-B*15:08, the HLA-B75 serotype marker with very low frequencies in Asian populations^10^,^12^. If an association between all HLA-B75 serotype members and CBZ-SJS is proven unequivocally, a screening policy for all HLA-B75 serotype will need to be considered in order to enhance the benefit of the *HLA-B* screening before CBZ prescription.

Furthermore, an *in silico* approach was performed to investigate the protein structure of the HLA-B75 members of interest, with respect to the reported HLA-B*15:01 structure as well as the molecular mechanism of CBZ in regard to the HLA-B75 members. Looking into the tertiary structure, the molecular dynamics (MD) simulation illustrated structural similarity among the antigen-presenting domain, except for HLA-B*15:21 (Fig. 2). Our findings suggest that the hypothesis that altered amino acids could affect the structure of the antigen-presenting domain in HLA-B75 serotype members and thus eventually lead to different CBZ binding affinities can be discarded. Instead, it appears that the selectivity of CBZ may be due to a different amino acid side chain.

Aside from the protein structure, the mechanism of CBZ when interacting with the HLA-B75 members is of interest, and it was for this reason the binding information was assessed via the molecular docking approach. In addition to HLA-B*15:02 previously demonstrated to have binding affinity to the CBZ molecule^13^,^14^, the molecular docking study revealed that HLA-B*15:11, HLA-B*15:21, and HLA-B*15:08 are also capable of binding to the CBZ molecule directly. In this study, we observed the three structure most capable of binding possible CBZ structures in the proposed CBZ-protein complex, on the basis of binding energy, so that we could evaluate the possible CBZ binding location.

Our study showed that CBZ bound an HLA-B*15:01, but in this study, the binding took place at a groove not participating in the antigen-presenting domain (Fig. 3). Since the HLA-B molecule has to function via a complex formation with the T-cell receptor using amino acids at an antigen-presenting area as a molecular interface, the presence of CBZ on other sites but this domain could play no effect on the induction of immune response.

On the other hand, CBZ was found to bind the HLA-B75 proteins using amino acids at the antigen-presenting area (Fig. 4). In most cases, namely HLA-B*15:02, B*15:08, and B*15:21, the different possible CBZ position could play a role at the protein interface between the HLA-B75 members and the T-cell receptor^14^. This suggests an action of CBZ at the atomistic level on some HLA-B75 protein members. To note about HLA-B*15:11, even though the best binding, with a relative energy of −6.82 KCal mol^−1^, indicates the CBZ may not affect the antigen-presenting area. An integration of CBZ on the antigen-presenting area of HLA-B*15:11 was raised by the second best binding with a relative of −6.18 KCal mol^−1^. Since these energies were somewhat close, these two CBZ positions could be possible. The former, the position (1) in Fig. 4c, can be found more though, and HLA-B*15:11-mediated T cell activation due to CBZ could be in a lesser extent, compared to the above-mentioned HLA-B proteins.

In conclusion, this report provides an additional CBZ-SJS case with HLA-B*15:21, but no HLA-B*15:02, to raise awareness of possible CBZ-SJS in patients who have negative screening for HLA-B*15:02. In addition, the study also provides the first evidence of a positive association between HLA-B*15:21 and CBZ-SJS along with molecular information regarding how CBZ reacts to the HLA-B75 family.

## Methods

### Pooled-data analysis

In this study, we performed a pooled-data analysis to evaluate the association between the uncommon members of the HLA-B75 serotype and CBZ-SJS, in the absence of cases and controls with HLA-B*15:02. We reviewed all studies listed in the HLA Adverse Drug Reaction Database which is a part of the Allele Frequencies Net Database (accessed on 3 June 2016)^10^. Full text and supplementary data of all case-control studies evaluating the association of *HLA-B* alleles and antiepileptic drug-induced severe cutaneous drug reactions were reviewed. The study inclusion criteria for pooling data included: (1) studies in Asian populations other than Japanese and Korean, (2) reported in English language, (3) *HLA-B* genotype, in high resolution, available in all CBZ-SJS cases, and (4) allele frequencies of HLA-B75 serotype members available in a tolerant control group. Only CBZ-SJS cases and CBZ tolerant controls were included in the pooled-data analysis. Thereafter, all cases and controls with HLA-B*15:02 alleles were excluded from the pooled data.

We analysed the statistical significance of the association between HLA-B75 serotype members and CBZ-SJS, using MedCalc (www.medcalc.org/calc/odds_ratio.php). The study protocol, including the case report, pooled-data analysis, and *in silico* analysis, was approved by the Institutional Research Ethics Committee, Faculty of Medicine, Prince of Songkla University (REC 59-229-05-1). For the case report, we obtained informed consent from the patient and his parents prior to collecting clinical information. The methods were carried out in accordance with the approved guidelines and relevant regulations.

### *In silico* analysis

A structure of the protein HLA-B75 family (HLA-B*15:02, B*15:08, B*15:11, and B*15:21) was built based on an HLA-B*15:01 crystal structure (pdb code 1XR9)^11^ as a molecular template. The homology modelling of the HLA-B75 family, except for HLA-B*15:21, was performed using the Rosetta Backrub online web server^15^. In addition, the secondary structure of the protein was independently predicted using the Psipred^16^ and NetSurfP^17^ web server tools to facilitate the selection of the best structure among 20 possible conformations. The best structure was chosen based on a scoring method and secondary structure prediction results. An HLA-B*15:21 model was constructed using Tleap module in the AMBER16 package^18^ to model serine-to-cysteine residue changes (Fig. 1). All missing hydrogen atoms were added into the chosen protein structure using the AMBER force field and a protonation state of all ionisable side chains was evaluated at pH 7.0 using the PDB2PQR online tool^19^,^20^. All structures were written in the RCSB Protein DataBank (PDB) format.

Molecular dynamic (MD) simulation was performed to emulate a fluctuated protein conformation. An implicit solvent model (Generalized Born/Surface Area, GBSA) was exploited to mimic the solvent condition. First, the structure was energy-minimised using the steepest descent method for 1,000 steps and then switched to a conjugate gradient method for 1,000 steps. Second, the minimised structure was sampled at 310 K (37 °C) regulated by a weak-coupling algorithm under an implicit 0.1 M NaCl solution. The MD simulation was carried out with the PMEMD module for 100 ns using a time step of 2 fs. The first 70 ns trajectory was omitted and the 300 equidistant snapshots from the last 30 ns trajectory were taken for a configuration average and conformation analysis.

In the molecular docking study, a CBZ structure was adopted from the PubChem Database, CID 2554. Ten HLA-B75 structures from the MD trajectory were chosen as “receptors” for CBZ. Both CBZ and the protein structures were written in the PDBQT format using the AutoDock Auxiliary Tool (ADT)^21^ based on the Autodock4 Universal Force Field (UFF). The study was first performed via Autogrid4, to cover an antigen-presenting area as a possible binding site, and Lamarckian genetic algorithm sampling implemented in Autodock4 yielded 100,000 possible drug-protein structures equal to (10 protein receptors) * (50 Genetic Algorithm runs) * (a population size of 200). The proposed structure was selected on the basis of lowest binding energy. All sampling variables were set as an Autodock4 default parameter unless stated otherwise.

## Additional Information

**How to cite this article:** Jaruthamsophon, K. et al. HLA-B*15:21 and carbamazepine-induced Stevens-Johnson syndrome: pooled-data and *in silico* analysis. *Sci. Rep.* **7**, 45553; doi: 10.1038/srep45553 (2017).

**Publisher's note:** Springer Nature remains neutral with regard to jurisdictional claims in published maps and institutional affiliations.

## Figures and Tables

**Table 1:** Carbamazepine-induced Stevens-Johnson syndrome (CBZ-SJS) cases and tolerant controls without HLA-B*15:02.

Study (Region)	CBZ-SJS cases			Tolerant controls	
	Total	Cases without HLA-B*15:02	*HLA-B* genotype of cases without HLA-B*15:02^a^	Total	Controls without HLA- B*15:02
Tassaneeyakul, 2010 (Thailand)^3^	42	5	(1) *18:01*/*56:04*(2) *15:21*/*48:01*(3) *27:06*/*56:01*(4) *15:21*/*40:01*(5) *15:11*/*46:01*	42	37
Cheung, 2013 (Hong Kong)^4^	26	2	(1) *13:01*/*54:01*(2) *13:01*/*15:01*	135	119
Khor, 2014 (Malaysia)^5^	5	3	(1) *15:08*/*56:01*(2) *15:01*/*41:01*(3) *40:06*/*51:06*	52	50
Nguyen, 2015 (Vietnam)^6^	35	3	(1) *13:01*/*46:01*(2) *51:02*/*54:01*(3) *38:02*/*51:02*	25	19
Total	108	13		254	225

The data was obtained from available studies in Asian populations with known associations between HLA-B*15:02 and CBZ-SJS.

^a^Underlined alleles represented members of the HLA-B75 serotype.

**Table 2:** Pooled-data analysis for associations between HLA-B*15:21 and B*15:11 and carbamazepine-induced Stevens-Johnson syndrome (CBZ-SJS) in cases without HLA-B*15:02.

Cases without HLA-B*15:02	CBZ-SJS cases without B*15:02 (n = 13)	Tolerant controls without B*15:02 (n = 225)	Odds ratio (95% CI), *P*-value
B*15:21	2	1	40.73 (3.43–484.22), *P* = 0.003
No B*15:21	11	224	
B*15:11	1	0^a^	54.12 (2.10–1397.09), *P* = 0.016
No B*15:11	12	225	

The data were obtained from 4 studies with high-resolution HLA-B typing results.

^a^As zero is present in the table, 0.5 was added to all cells before calculating the odds ratios.

**Table 3:** Binding energy among CBZ and HLA-B*15: 01 and HLA-B75 protein members.

HLA-B molecule	Possible observed binding energy (Kcal mol^−1^)
HLA-B*15:01	−7.14
HLA-B*15:02	−7.89
HLA-B*15:08	−7.10
HLA-B*15:11	−6.82, −6.18
HLA-B*15:21	−6.61

Molecular docking was performed between CBZ and ten possible protein structures of each HLA-B molecule obtained from an MD simulation. The binding energy was calculated for each case of CBZ bound to the antigen-presenting area of the protein.
